# Association between elevated serum transaminase and moderately increased albuminuria: a cross-sectional study in western Tokushima, Japan

**DOI:** 10.1186/s12882-023-03411-y

**Published:** 2023-12-05

**Authors:** Shoichi Fukuda, Ukyo Shirase, Shigeru Ogimoto, Mai Nakagawa, Kazumi Nakagawa, Ayumu Tominaga, Hisayoshi Morioka

**Affiliations:** 1 Department of Public Health, Graduate School of Biomedical Sciences, Tokushima University, 3-18-15, Kuramoto, Tokushima city, Tokushima 770-8503 Japan; 2 Mima Public Health Centre, 23-23, Myouren, Anabuki, Mima city, Tokushima 777-0005 Japan

**Keywords:** Moderately increased albuminuria, Chronic Kidney Disease, Liver disorder, Fatty liver, Epidemiology

## Abstract

**Background:**

This study aimed to identify the factors relating to moderately increased albuminuria among middle-aged and older individuals in Japan.

**Methods:**

We conducted specific health examinations in which we measured albuminuria levels, and administered a questionnaire survey to record participants’ lifestyles in western Tokushima Prefecture, Japan. A total of 1,660 people whose albuminuria was less than 300 mg/g creatinine (Cr) were analyzed. We divided participants into two groups—those with normal albuminuria (< 30 mg/gCr) and those with moderately increased albuminuria (≥ 30 mg/gCr, > 300 mg/gCr)—and compared their characteristics. To investigate all relevant factors, we conducted a multivariate logistic regression analysis.

**Results:**

The moderately increased albuminuria group were significantly older and had, among them, significantly higher percentages of a body mass index (BMI) ≥ 25 kg/m^2^, diabetes, hypertension, and mild liver disorder (aspartate transaminase ≥ 31 U/L or alanine aminotransferase ≥ 31 U/L or gamma-glutamyl transferase ≥ 51 U/L). (*p* < 0.01) In a multivariate logistic regression analysis that used microalbuminuria as an independent variable, we found the adjusted odds ratio (AOR) and 95% confidence interval (CI) to be significantly higher among individuals with diabetes (AOR: 2.04, 95% CI: 1.40–2.99); hypertension (AOR: 1.90, 95% CI: 1.36–2.65); BMI ≥ 25 kg/m^2^ (AOR: 1.76, 95% CI: 1.27–2.44); and mild liver disorder (AOR: 1.54, 95% CI: 1.10–2.18).

**Conclusions:**

In addition to diabetes, hypertension, and BMI ≥ 25 kg/m^2^, this study found that among the middle-aged and older general population living in western Tokushima Prefecture, there were cases of mild liver disorder (elevated serum transaminase), which independently associated with moderately increased albuminuria. Therefore, in health checkups targeting the general population, there is a need to consider measuring albuminuria, even in those who have only mild liver dysfunction (health guidance level).

**Trial registration:**

N/A.

## Background

Patients with chronic kidney disease (CKD) mainly present with reduced glomerular filtration rates (GFR; < 60 mg/g creatinine [Cr)) and/or symptoms of albuminuria [1]. Those whose CKD has progressed to end-stage renal disease undergo renal dialysis and/or receive renal transplantation [2]. Previous studies showed CKD was associated with cardiovascular disease [3], stroke [4], severe anemia [5], and other diseases, as well as a rise in mortality rates [6]. The number of CKD patients worldwide is growing, and the World Health Organization predicts that the CKD mortality rate per 100,000 people will increase from 12.2 in 2012 to 14 in 2030 [7]. In Japan, the number of CKD patients is estimated, to be approximately 13.3 million, or one out of roughly eight people aged 20 and older [8]. CKD is one of the most serious public health problems globally.

Moderately increased albuminuria refers to more than 30 mg/gCr, but less than 300 mg/gCr [9]. We know that diabetic nephropathy can be detected early in patients with diabetes by measuring microalbuminuria levels [10]. Therefore, regular tests for urinary albumin in diabetes patients are available in Japan and covered by public medical insurance. A previous population-based cohort study reported that moderately increased albuminuria is a predictor of death caused by cardiovascular disease [11]. Urinary albumin levels are considered an indicator of vascular endothelial damage, including renal glomerular and cardiovascular damage.

Therefore, if urinary albumin tests are conducted in health checkups for the general population and appropriate measures are taken for those with moderately increased albuminuria, such as visits to nephrologists and health guidance for lifestyle modification, atherosclerotic diseases, including end-stage renal disease and cardiovascular disease, may be prevented. However, health checkups for individuals in Japan generally do not include albuminuria tests.

Previous epidemiological patient-based or hospital-based studies involving patients with diabetes have reported that moderately increased albuminuria is a predictor of end-stage renal disease and decreasing estimated GFR (eGFR) [12]. These studies have also reported that moderately increased albuminuria is associated with high blood pressure [13, 14] and elevated body mass index (BMI) [14, 15]. However, few population-based epidemiological studies have reported on moderately increased albuminuria and its other associated factors. In the general population, moderately increased albuminuria has been reported to be associated with high fasting blood sugar [13], high blood pressure [13] and elevated BMI [15]. Especially, to our knowledge, there exist no population-based epidemiological studies that have reported an association between moderately increased albuminuria and elevated serum transaminase. If the association between moderately increased albuminuria and elevated serum transaminase is clarified in the general population, atherosclerotic diseases and may be prevented more effectively in health checkups.

Therefore, this study aimed to identify various associated factors including moderately increased albuminuria and elevated serum transaminase. In western Tokushima Prefecture, Japan, we measured the albuminuria levels of the residents recruited for this study who were receiving health checkups.

## Methods

### Study participants

The study participants were residents of the cities of Mima and Miyoshi and the towns of Tsurugi and Higashi-Miyoshi, in western Tokushima Prefecture. They underwent regular specific health checkups at various health care centers. These specific health checkups are offered each year to individuals aged 40–74 to prevent or improve metabolic syndrome.

In preparation for the study, the authors provided an explanatory leaflet to these health care centers, as well as verbal explanations regarding the survey to individuals undergoing specific health checkups. In addition to undergoing various clinical tests and answering the questions that form part of the specific health checkups, those who had agreed to participate in the survey had their urinary albumin measured.

In total, 1,693 people took part in the survey from June 2021 to January 2022. There were no participants with missing data, and a total of 1,660 participants—after excluding those with macroalbuminuria—were analyzed. The characteristics of the analyzed participants are shown in Table 1.

**Table 1:** Characteristics of the analyzed participants (*N* = 1,660)

	N	%
**Sex**		
Male	725	43.7
Female	935	56.3
**Age**		
40–49 years old	110	6.6
50–59 years old	126	7.6
60–69 years old	771	46.4
70–74 years old	653	39.3
**Body mass index**		
≥ 25 kg/m^2^	430	25.9
< 25 kg/m^2^	1,230	74.1
**Currently smoking**		
Yes	174	10.5
No	1,486	89.5
**Moderate drinking**		
Yes	1,401	84.4
No	259	15.6
**Habitual exercise**		
Yes	770	46.4
No	890	53.6
**Diabetes**		
Diabetes	206	12.4
Normal, Borderline	1,454	87.6
**Hypertension**		
Hypertension	773	46.6
Normal	887	53.4
**Dyslipidemia**		
Hypertension	962	58.0
Normal	698	42.0
**Albuminuria**		
Moderately increased albuminuria (≥ 30 mg/gCr and < 300 mg/Cr)	204	12.3
Normoalbuminuria(< 30 mg/gCr)	1,456	87.7
**Aspartate transaminase(AST)**		
≥ 31U/L	204	12.3
< 31U/L	1,456	87.7
**Alanine transaminase(ALT)**		
≥ 31U/L	204	12.3
< 31U/L	1,456	87.7
**Gamma-glutamyl transferase(GGT)**		
≥ 51U/L	201	12.1
< 51U/L	1,459	87.9
**Mild liver disorder**		
AST ≥ 31U/L or ALT ≥ 31U/L or GGT ≥ 51U/L	382	23.0
AST < 31U/L and ALT < 31U/L and GGT < 51U/L	1,278	77.0

To protect the participants’ personal information, we eliminated their names and dates of birth from all their data. This study was conducted in accordance with the Declaration of Helsinki and the national ethical guidelines. Informed consent was obtained from each participant. This study protocol was approved by the Ethics Committee of Tokushima University Hospital (approval number: 3944).

### Measurements

Clinical tests performed during specific health checkups included the following: height (m), weight (kg), systolic and diastolic blood pressure (mmHg), fasting blood glucose (tested after fasting for at least ten hours; mg/dl), HbA1c (%), LDL cholesterol (mg/dl), HDL cholesterol (mg/dl), triglycerides (mg/dl), serum aspartate aminotransferase (AST; U/l), serum alanine aminotransferase (ALT; U/l), and serum gamma-glutamyl transferase (GGT; U/l).

The questionnaire provided during the specific health checkups included the following items: smoking and drinking habits, medication history (antihypertensive drugs, diabetes drugs, dyslipidemia drugs), and tendency to partake in habitual exercise.

BMI was calculated using the formula BMI (kg/m^2^) = Weight (kg)/ Height (m)× Height (m), and any values exceeding 25 kg/m^2^ were seen as indicating obesity. BMI of less than 25 kg/m^2^ was considered not obesity.

Regarding smoking, the answer was set as two options, “Yes” or “No,” to the question, “Are you currently a habitual smoker?” (Previous habitual smokers who had refrained from smoking for the past month or more were instructed to answer “No”). Participants who answered “Yes” were placed in the category of “Currently smoking: Yes.” Regarding drinking, three options (“Every day,” “Occasionally,” and “Sometimes”) were set as the answers to the question: “How often do you drink alcohol beverages per week?” For the question asking about the drinking quantity—“How much alcohol beverages do you drink per day?”—four options were set (“1 drink/day,” “1–2 drinks/day,” “2–3 drinks/day,” and “3 or more drinks/day”). One drink is equivalent to approximately 20 g of ethanol consumption. Regarding “Currently drinking,” participants who met both criteria, in other words “drinking every day” and “drinking quantity exceeding 1 drinks/day,” were placed in the category of “Moderate drinking: No.”

Two options, “Yes” and “No”, were set for the question pertaining habitual exercise habits: “Have you been engaging in a sweat-inducing workouts lasting more than 30 minutes per session, more than twice per week for more than a year?” Participants who answered “Yes” were placed in the category of “Habitual exercise: Yes.”

Hypertension was defined as follows: systolic blood pressure ≥ 140 mmHg, diastolic blood pressure ≥ 90 mmHg, or currently taking antihypertensive medications [11]. In all other cases, it was assumed to be normal. Diabetes was defined as HbA1c ≥ 6.5%, fasting blood glucose ≥ 126 mg/dl, or currently taking antihyperglycemic medications or insulin injections [12]. In all other cases, it was assumed to be normal and borderline. Dyslipidemia was defined as LDL cholesterol ≥ 140 mg/dl, HDL cholesterol < 40 mg/dl, Triglycerides ≥ 150 mg/dl, or currently taking dyslipidemia medication [11]. In all other cases, it was assumed to be normal. Mild liver disorder was defined as follows, based on standard specific health checkups and the health guidance program formulated by the Japanese Health Ministry in 2018: AST ≥ 31 IU/L, ALT ≥ 31 IU/L, or GGT ≥ 51 IU/L [16]. In Japan, this is the level of liver damage which signals the need for health intervention and so on. In all other cases, it was assumed to be normal.

Albuminuria, according to a midstream spot urine test, was measured using immune nephelometry. Based on the Japanese Society of Nephrology’s guidelines, we divided albuminuria into three categories: normoalbuminuria (< 30 mg/gCr), moderately increased albuminuria (≥ 30 mg/gCr and < 300 mg/gCr) (formerly called albuminuria), and macroalbuminuria (≥ 300 mg/gCr) [17].

### Statistical analysis

First, the participants’ background characteristics were compared between the normoalbuminuric group and the moderately increased albuminuria group. Continuous variables were shown using the median and standard deviation, while categorical variables were shown using proportion (%). To confirm the differences between the two groups, the Student’s *t*-test and the chi-squared test were used.

Second, to calculate the adjusted odds ratio (AOR) and 95% confidence intervals (CIs) for the moderately increased albuminuria with elevated serum transaminase, the multivariate logistic regression model was applied. The model was adjusted for mild liver disorder as well as for sex, age, BMI, currently smoking, moderate drinking, habitual exercise, diabetes, and hypertension. Previous population-based epidemiological studies found an association between moderately increased albuminuria and diabetes, hypertension and obesity. (Model 1)

Third, to calculate the AOR and 95% CIs for the association of moderately increased albuminuria with the combination of elevated AST or low AST/ALT ratio, and elevated GGT, the multivariate logistic regression model was applied. In Model 2, a combination of elevated AST and elevated GGT was added in place of the mild liver disorder of Model 1. In Model 3, a combination of low AST/ALT ratio and elevated GGT was added in place of the mild liver disorder of Model 1.

Statistical tests were based on two-side probabilities, and a *p* value of less than 0.05 was considered significant. All statistical analyses were performed using IBM SPSS Statistics version 28.0 for Windows.

## Results

Table 2 shows the differences between the normoalbuminuric group and the moderately increased albuminuria group. Compared with the normoalbuminuric group, the moderately increased albuminuria group were, in general, significantly older, and had a significantly higher percentage of participants with BMI ≥ 25 kg/m^2^, diabetes, hypertension, dyslipidemia, and mild liver disorder (AST ≥ 31 U/L or ALT ≥ 31 U/L or GGT ≥ 51 U/L). (*p* < 0.001)

**Table 2:** Characteristics of normoalbuminuric and moderately increased albuminuria groups

		Normoalbuminuria		Moderately increased albuminuria		
		N = 1,456(87.7%)		N = 204(12.3%)		*P* Value
Age (year)		65.7(7.9)		67.8(6.0)		0.001
Sex(male)(%)		42.9		49.5		0.073
BMI ≥ 25 kg/m^2^(%)		23.8		41.2		< 0.001
Currently smoking (%)		10.2		12.3		0.377
Moderate drinking (%)		84.5		83.3		0.655
Habitual exercise (%)		46.7		44.1		0.488
Diabetes (%)		10.7		24.5		< 0.001
Hypertension (%)		43.8		66.7		< 0.001
Dyslipidemia(%)		57.5		61.3		0.305
AST ≥ 31U/L(%)		11.3		19.1		0.002
ALT ≥ 31U/L(%)		11.1		20.6		0.001
GGT ≥ 51U/L(%)		11.4		17.2		0.018
Mild liver disorder (%)		21.4		34.3		< 0.001

Data are presented as means ± standard deviation or percentages

For calculation of the *P* values, Student’s t-test and chi-squared test were used

Mild liver disorder: Participants who had one or more values (AST ≥ 31U/L or ALT ≥ 31U/L or GGT ≥ 51U/L) were categorized as having mild liver disorder

Abbreviations: BMI, body mass index, AST, aspartate transaminase; ALT, alanine transaminase; GGT, gamma-glutamyl transferase

Table 3 shows the results of the multivariate logistic regression analysis regarding associations with moderately increased albuminuria. (Model 1) The AOR for moderately increased albuminuria was significantly higher among patients with mild liver disorder (AST ≥ 31 U/L or ALT ≥ 31 U/L or GGT ≥ 51 U/L; AOR: 1.54, 95% CI: 1.10–2.18). Other AORs for moderately increased albuminuria were diabetes (AOR: 2.04, 95% CI: 1.40–2.99), hypertension (AOR: 1.90, 95% CI: 1.36–2.65), and BMI ≥ 25 kg/m^2^ (AOR: 1.76, 95% CI: 1.27–2.44).

**Table 3:** Multivariate logistic regression analysis regarding associations on moderately increased albuminuria

	Moderately increased albuminuria(N = 204)					
**Model 1**	N	AOR	95%CI			P for trend
**Mild liver disorder**						0.013
No	1,278	1.00	Reference			
Yes	382	**1.54**	**1.10**	**-**	**2.18**	
**Sex**						0.855
Male	725	1.00	Reference			
Female	935	1.03	0.73	-	1.46	
**Age**						0.033
40–49 years	110	1.00	Reference			
50–59 years	126	1.41	0.50	-	3.97	
60–69 years	771	1.77	0.73	-	4.25	
70–74 years	653	**2.53**	**1.05**	-	**6.13**	
**BMI**						0.001
< 25 kg/m^2^	1,230	1.00	Reference			
≥ 25 kg/m^2^	430	**1.76**	**1.27**	**-**	**2.44**	
**Currently smoking**						0.681
No	1,486	1.00	Reference			
Yes	174	1.11	0.68	-	1.82	
**Moderate drinking**						0.560
Yes	1,401	1.00	Reference			
No	259	0.88	0.56	-	1.37	
**Habitual exercise**						0.157
Yes	770	1.00	Reference			
No	890	1.25	0.92	-	1.71	
**Diabetes**						< 0.001
Normal, Border line	1,454	1.00	Reference			
Diabetes	206	**2.04**	**1.40**	**-**	**2.99**	
**Hypertension**						< 0.001
Normal	887	1.00	Reference			
Hypertension	773	**1.90**	**1.36**	**-**	**2.65**	

For calculation of the *P* values, a multivariate analysis model (forced entry method) was used

Mild liver disorder: Participants who had one or more values (AST ≥ 31U/L or ALT ≥ 31U/L or GGT ≥ 51U/L) were categorized as having mild liver disorder

Abbreviations: BMI, body mass index, AST, aspartate transaminase; ALT, alanine transaminase; GGT, gamma-glutamyl transferase; AOR, adjusted odds ratio; CI, confidence interval

Table 4 shows the results of the multivariate logistic regression analysis regarding associations of moderately increased albuminuria combined with elevated ALT or low AST/ALT ratio and elevated GGT. In Model 2, compared with those with ALT < 31IUL and GGT < 31IU/L, there was a non-significant increase in AOR in the other groups. In Model 3, compared with those with ALT < 31IUL and GGT < 31IU/L, there was a significant increase in AOR in those with AST/ALT < 1 and GGT < 51IU/L (AOR: 1.52, 95% CI: 1.02–2.27).

**Table 4:** Association of combination of AST or AST/ALT ratio, and GGT with moderately increased albuminuria

Moderately increased albuminuria (N=204)													
	Model 2							Model 3					
	N	AOR	95%CI			P for trend		N	AOR	95%CI			P for trend
						0.231							0.098
ALT < 31IU/L and GT < 51IU/L	1,337	1.00	Reference				AST/ALT ≥ 1 and GGT < 51IU/L	1,186	1.00	Reference			
ALT < 31IU/L and GGT ≥ 51IU/L	119	1.27	0.71	-	2.27		AST/ALT ≥ 1 and GGT ≥51IU/L	118	1.58	0.89	-	2.82	
ALT ≥ 31IU/L and GGT < 51IU/L	122	1.39	0.83	-	2.33		AST/ALT < 1 and GGT < 51IU/L	273	**1.52**	**1.02**	**-**	**2.27**	
ALT ≥ 31IU/L and GGT ≥ 51IU/L	82	1.72	0.95	-	3.12		AST/ALT < 1 and GGT ≥ 51IU/L	83	1.59	0.85	-	2.97	

For calculation of the *P* values, multivariate analysis model was used

Adjusted for sex, age, BMI, currently smoking, moderate drinking, habitual exercise, diabetes, hypertension

Abbreviations: AST, aspartate transaminase; ALT, alanine trans aminase; GGT, gamma-glutamyl transferase; AOR, adjusted odds ratio; CI, confidence interval

## Discussion

The results of our population-based cross-sectional study shows an association between moderately increased albuminuria and diabetes, hypertension, obesity and mild liver dysfunction (health guidance level), independently. Mild liver disorder (health guidance level) is seen as being present when one or more of the following are met: AST ≥ 31 U/L or ALT ≥ 31 U/L or GGT ≥ 51 U/L. Mild liver disorder refers to participants who show normal-high serum transaminase. In this study, the percentage of mild liver disorder was 23.0%, while that of the general population was rather high. To our knowledge, few population-based studies have shown that moderately increased albuminuria is independently associated with obesity/liver disorder. More specifically, our study is the first to illustrate the association between moderately increased albuminuria and mild liver disorder (health guidance level) independently. Our results suggest that, in health checkups geared toward the general population, testing albuminuria be considered, even in those who have only mild liver disorder (health guidance level).

Previous epidemiological studies showed that proteinuria, or CKD including proteinuria (≥ (1+), dipstick analysis), not moderately increased albuminuria, or decreasing eGFR is associated with elevated serum GGT or ALT. Ishigami et al. addressed the independent association between proteinuria and elevated serum GGT in their population-based cross-sectional study [18]. Moreover, Shen et al. reported that elevated serum GGT was a predictor of the incidence of CKD in their population-based cohort study [19]. Further of note here is the work from Ochiai et al., who—following their study, which included middle-aged women who had an annual health checkup—stated that elevated serum ALT was associated with CKD, regardless of GGT elevation [20].

In recent years, population-based studies have reported an association between non-alcoholic fatty liver disease (NAFLD) and CKD [21, 22]. Indeed, an epidemiological study focused on prediabetic and non-diabetic hospitalized patients revealed that NAFLD, but not mild liver disorder, was independently associated with moderately increased albuminuria [23]. However, to our knowledge, in a population-based study, moderately increased albuminuria has not been associated with mild liver disorder such as elevated serum GGT, ALT, and AST. From earlier stages of metabolic syndrome, mild renal abnormalities may be associated with mild liver disorder.

A large-scale Japanese cross-sectional study showed the lowest probability of proteinuria in mild drinkers. [18] The same study suggested that GGT had a clinically greater impact on proteinuria than alcohol consumption [18]. This study found no significant association between moderately drinking and moderately increased albuminuria. However, the association with elevated GGT was similar. Future studies should conduct a survey that includes the amount and frequency of different types of alcoholic beverages and drinking patterns.

Given the mechanism of association between liver disorder and renal diseases such as moderately increased albuminuria, decreasing eGFR and so on, we are assuming increased oxidative stress and insulin resistance. Oxidative stress is known to cause proteinuria [24] and lead to increase serum GGT levels [25]. Serum GGT levels have a potential use as a marker of oxidative stress. Moreover, serum GGT is reported to be associated with insulin resistance [26], while insulin resistance is known to be associated with renal abnormalities such as CKD, for example [27]. More specifically, an epidemiological study among non-obese and non-diabetic inpatients showed that moderately increased albuminuria was independently associated with insulin resistance [28].

Elevated serum ALT was found to be independently associated with CKD among middle-aged women, regardless of the GGT elevation [20]. A cross-sectional study in a rural Chinese population revealed a positive association between elevated serum ALT levels and cardiometabolic risk factors, such as hypertension and abdominal obesity [29]. Elevated serum ALT may be caused by damaged liver cells and the accumulation of fat in the liver. However, the mechanism of association between elevated serum ALT and renal disease remains unclear.

The present study showed that low AST/ALT ratio was significantly associated with moderately increased albuminuria even in the absence of elevated GGT. However, there was no significant association between elevated ALT and increased albuminuria. (Table 4) Further studies are needed to clarify the association between the combination of elevated AST or low AST/ALT ratio, elevated GGT, and moderately increased albuminuria.

This study has several limitations that must be acknowledged. First, we were not able to determine the causal relationship between moderately increased albuminuria and liver disorder (health guidance level) because our study was cross-sectional in nature. Second, the data on smoking, drinking, exercise, and so on were based on reports compiled by the respondents themselves, so recall bias may exist; participants tended to deny that they smoked, so there was a risk of under-reporting. Indeed, a study which targeted the middle-aged and elderly population showed smoking to be independently associated with moderately increased albuminuria [30]. In the above-mentioned survey, smokers and non-smokers were identified based on the concentrations of urinary cotinine. Third, as the present study included participants who had already taken part in regular group health checkups, there was a risk that a selection-bias may have existed. There was also a likelihood that the sample included a large number of highly health-conscious people. People who regularly visit clinics may have received their health-checkups there. Indeed, in general, health checkup rates were not very high. In Japan, the national participation rate for specific health checkups was 53.1% in FY2021 [31]. Fourth, there is a possibility that confounding factors existed, other than those which were adjusted in the present study. As we conducted no oral glucose tolerance tests, borderline diabetes was not included as a covariate. High normal blood pressure, which was similarly not included as a covariate, is also reported to be associated with moderately increased albuminuria [13].

## Conclusions

This study recruited middle-aged and older general residents as participants and showed that an independent association exists between elevated serum transaminase and moderately increased albuminuria. As such, in health checkups targeting the general population, there is a need to consider measuring albuminuria, even in individuals who have only mild liver disorder (health guidance level).

## Data Availability

The datasets analyzed during the current study are available from the corresponding author on reasonable request, and have been approved by the Ethics Committee of Tokushima University Hospital.

## Abbreviations

CKD: chronic kidney disease
GFR: glomerular filtration rates
Cr: creatinine
eGFR: estimated GFR
BMI: body mass index
AST: aspartate transaminase
ALT: alanine transaminase
GGT: gamma-glutamyl transferase
AOR: adjusted odds ratio
CI: confidence interval
NAFLD: non-alcoholic fatty liver disease
